# Spatial epidemiological analysis based on township scale and analysis of influencing factors of pulmonary tuberculosis cure of Changshu city from 2015 to 2022

**DOI:** 10.1371/journal.pone.0317269

**Published:** 2025-01-16

**Authors:** Xiao-yan Xu, Zheng-yuan Zhou, Li-qiang Gong, Li-qiang Xu, Xiao-kang Jiao, Bian Yin, Tian-hong Jiang

**Affiliations:** 1 Infectious Disease Prevention Section, Changshu Center for Disease Control and Prevention, Changshu, Jiangsu, China; 2 Office of the Director, Changshu Center for Disease Control and Prevention, Changshu, Jiangsu, China; 3 Computer Science, Yiducloud (Beijing) Technologies Co., Beijing, China; 4 Biostatistics, Yiducloud (Beijing) Technologies Co., Beijing, China; 5 Information Technology Section, Changshu Center for Disease Control and Prevention, Changshu, Jiangsu, China; Hangzhou Red Cross Hospital, CHINA

## Abstract

**Objective:**

This study aimed to enhance the prevention and control of pulmonary tuberculosis (PTB) and provide more effective and accurate methods in Changshu City.

**Methods:**

The PTB patients’ information came from the China Information System for Disease Control and Prevention (CISDCP). The demographic data for Changshu city and towns came from the Suzhou Statistical Yearbook and the LandScan platform. ArcGIS was used for global spatial autocorrelation analysis and local spatial autocorrelation analysis. Univariate logistic regression and multivariate logistic regression were used to analyze the influencing factors of cured PTB patients. The receiver operating characteristic (ROC) curve and decision curve analysis (DCA) were used to analyze the predictive efficacy and clinical benefit of the indicators. XGBoost analysis was performed to explore the feature importance of key metrics for PTB outcome.

**Results:**

A total of 3943 PTB cases were included. The annual incidence rate of new PTB in Changshu city was 27.081 per 100,000. Changshu High-tech Industrial Development Zone in Jiangsu Province and Shajiabang town were the high-high aggregation areas and hot spot areas. Diagnosis delay, TB strain types, and drug sensitivity were independent predictors of the cure of new PTB patients.

**Conclusion:**

The central and southern areas of Changshu were the high-high cluster areas and hot spots for PTB. Shorter diagnosis delay days and mycobacterium tuberculosis (MTB) promote the cure of tuberculosis, while drug sensitivity was a risk factor for its cure.

## 1. Introduction

Tuberculosis (TB) is a chronic and infectious disease caused by mycobacterium tuberculosis (MTB) infection. It is a major public health problem that affects millions of people worldwide. According to the WHO Global Health Estimates, TB was the main cause of death in low-income countries and middle- and low-income countries, ranking eighth and seventh, respectively [1]. The incidence rate of TB globally was 133 per 100,000 while in China it was 52 per 100,000 [2]. Pulmonary tuberculosis (PTB) is the primary type of TB, accounting for approximately 85% of all cases. PTB ranked second in the number of cases and deaths among legally reported category A and B notifiable infectious diseases in China. In 2020, China reported 670,538 cases of PTB with an incidence rate of 47.7644 per 100,000. The disease resulted in 1919 deaths, corresponding to a mortality rate of 13.67 per 100,000. During the same year, there were 28.4039 cases of PTB per 100,000 in Jiangsu Province, with a total of 22,922 cases deaths [3].

Spatial epidemiology is a field of epidemiology that utilizes spatial analysis technology and geographic information systems to describe and analyze the development, change, and spatial distribution characteristics of population health events, health, and disease [4]. At present, spatial epidemiological analysis methods have been widely used in the study of infectious diseases, chronic non-communicable diseases, and many other diseases and related factors. The main methods of spatial epidemiology are spatial autocorrelation analysis, spatial clustering analysis, spatial interpolation method, and spatial regression model. Spatial autocorrelation refers to the correlation of the same variable at different spatial locations, which is a measure of the degree of cluster of spatial unit attribute values. Spatial autocorrelation analysis comprises global and local spatial autocorrelation analysis. Global Moran’s Index (Global Moran’s I) and Anselin Local Moran’s Index (Anselin Local Moran’s I) are the most used in global and local spatial autocorrelation analysis, respectively.

Changshu city is located in the southeast of Jiangsu Province. The incidence rate of PTB ranked first in the category A and B notifiable infectious diseases in Changshu city [5]. It was found that the incidence rate of PTB from 2005 to 2015 in Changshu city was between 26.96 per 100000 population to 41.99 per 100000 population [6]. At this stage, studies on the incidence of PTB had mainly focused on the provincial [7] and municipal levels [8], with only a few studies focusing on the township scales [9]. Few studies have focused on the incidence of PTB using spatial epidemiological methods and the influencing factors related to the cure of PTB patients in Changshu city in recent years. To enhance the prevention and control of PTB in Changshu city and provide more effective and accurate methods, we conducted a spatial epidemiological analysis of the incidence of PTB in Changshu city from 2015 to 2022 and analyzed the influencing factors of the cure of PTB patients.

## 2. Methods

### 2.1 Data sources and collected indicators

The PTB patients’ information from 2015 to 2022 was obtained from the China Information System for Disease Control and Prevention (CISDCP).

Patients’ information included age, sex, nation, occupation, key population, types of current address [local, other counties and districts in this city, other cities in this province, other provinces], types of domicile address (local, intra-city mobility, inter-city mobility (intra-provincial mobility), inter-provincial mobility), patient sources (referral, tracking, seeing a doctor directly, health check-up, others), case types (new cases, recurrent cases), body mass index (BMI), diagnostic delay, education, marital status, alcohol, smoke, TB strain types (MTB, non-tuberculous mycobacteria), drug sensitivity, comorbidities, using of fixed dose compound (FDC), 2HRZE4HR.

The data of the permanent population for Changshu city and the population data of Changshu towns from 2015 to 2022, were obtained from the Suzhou Statistical Yearbook (The Suzhou Municipal Bureau of Statistics [10], and the LandScan platform, respectively. The LandScan platform provides the highest resolution population distribution data on a global scale.

Diagnostic delay refers to the time from the first symptom of a patient to the diagnosis of PTB.

The key population includes HIV/AIDS patients, diabetics, school nursery staff, supervisors, breeders, dust workers/pneumoconiosis patients, mental hospital patients, nursing home residents, welfare home residents, close contacts of TB patients, and medical staff.

2HRZE/4HR is a common treatment scheme for the initial treatment of active PTB, that is, isoniazid (H), rifampicin (R), pyrazinamide (Z) and ethambutol (E) are used once a day for 2 months in the intensive period, and isoniazid and rifampicin are used once a day for 4 months in the subsequent consolidation period.

Referral: the medical institution transfers the patients treated in this unit to another medical institution for treatment or treatment according to the needs of the illness.

Tracing: the primary healthcare institution to assist the county-level disease prevention and control institutions in tracing patients with TB and suspected TB who have made outbreak reports but have not been to the designated TB healthcare institution for treatment.

See a doctor directly: the process in which patients go to hospitals, clinics, and other medical institutions in person for diagnosis, treatment, or consultation by doctors or medical professionals due to cough and expectoration.

Health check-up: physical check-up of the examinee through medical means and methods, so as to understand the health status of the examinee, early detection of disease clues, and diagnosis and treatment behaviors of health risks.

Others: TB found by means other than referral, tracking, see a doctor directly, health check-up.

### 2.2 Ethical statement

The study was approved by the Ethics Committee of Changshu Center for Disease Control and Prevention (Ethical approval number: CSCDC2022002). All patients who were familiar with the contents and processes of the study and able to complete all the scheduled study processes signed the informed consent. Our study complies with the Declaration of Helsinki.

### 2.3 Descriptive analysis

The time distribution of PTB patients in different subgroups under different clinical characteristics was counted. The time distribution of PTB was counted in years and months respectively.

### 2.4 Spatial autocorrelation analysis

ArcGIS 10.8.1 was used for global spatial autocorrelation analysis and local spatial autocorrelation analysis.

Global spatial autocorrelation analysis: Global Moran’s I is used to reflect the cluster degree of PTB incidence rate in the whole region. The range of the Global Moran’s Ⅰ is [–1,1], in which Ⅰ>0 means positive correlation; Ⅰ = 0 means that the region is randomly distributed and has no spatial correlation; Ⅰ<0 means negative correlation distribution [8,11].

local spatial autocorrelation analysis: Anselin Local Moran’s I was used to detect high-value cluster and low-value cluster in Changshu city. Getis-Ord *G* statistic is a measure of local spatial autocorrelation, and test its statistical significance with standardized statistic Z(G). When G>0, and *P*<0.05, it indicates that there is a high-value cluster in the study area; When G<0 and *P*<0.05, it indicates that there is a low-value cluster in the study area [12,13].

High-high cluster areas indicate that areas with high incidence have high incidence in neighboring areas.

Low-low cluster areas indicate that areas of low prevalence are adjacent to areas of low prevalence.

Low-high outlier areas indicate that areas of low prevalence are adjacent to areas of high prevalence.

High-low outlier areas indicate that areas of high prevalence are adjacent to areas of low prevalence.

Hot spot areas indicate that high-value clusters exist in this area.

Cold spot areas indicate that low-value clusters exist in this area.

### 2.5 Statistical analysis

The statistical analysis was conducted using R 4.3.2 software. The ggplot2 package was used to plot the distribution of PTB incidence and the number of PTB cases, the rmda package was used to plot DCA curves, the qROC package was used to plot ROC curves, and the XGboost package was used to perform the plotting of feature importance rankings. The measurement data were expressed by median (P25, P75) as they conformed to non-normal distribution, and the Mann-Whitney U test was used for analyzing the difference between the two groups. The counting data were expressed by frequency and percent, and the χ^2^ test was used for analyzing the distribution differences. According to whether PTB patients were cured or not, they were divided into cured group and not cured group. Univariate logistic regression and multivariate logistic regression analysis was used to analyze the independent factors of PTB cured. The receiver operating characteristic (ROC) curve and decision curve analysis (DCA) curve were used to analyze the predictive efficacy and clinical benefit of the indicators. XGBoost analysis was carried out to explore the order of feature importance of key indicators of PTB results. P<0.05 was considered that the difference is statistically significant.

## 3. Results

### 3.1 Patients’ basic information

The demographic and clinical profile of the patients were shown in **Table 1**. 3943 PTB patients in 2015–2022 from various townships in Changshu city were recruited in this study. The participants in the study were mainly between 19 and 59 years old, of whom 72.61% were male and 27.39% were female. The patients’ occupations were mainly farmers, workers, and household and non-working, and most of the people belonged to the Han nationality. Approximately 70% of patients were referred for treatment, with only a small number (170, 4.311%) seeing a doctor directly.

**Table 1:** Demographic and clinical profile of the patients in Changshu city, 2015–2022.

Variable		Number (%)
N		3943
Age	0–18	110 (2.790)
	19–59	2341 (59.371)
	60–100	1492 (37.839)
Sex	male	2863 (72.610)
	female	1080 (27.390)
Nation	Han nationality	3864 (97.996)
	others	79 (2.004)
Occupation	catering and food industry staff	29 (0.735)
	cadre staff	32 (0.812)
	workers	575 (14.583)
	attendants in public places	5 (0.127)
	household and non-working	484 (12.275)
	teachers	14 (0.355)
	retirees	345 (8.750)
	migrant worker	125 (3.170)
	farmers	1642 (41.643)
	business service workers	320 (8.116)
	students	101 (2.562)
	medical personnel	20 (0.507)
	fishermen	6 (0.152)
	others	245 (6.214)
Key populations	yes	291 (7.380)
	no	3652 (92.620)
Types of current address	local	3932 (99.721)
	other counties and districts in this city	1 (0.000)
	other cities in this province	3 (0.076)
	other provinces	7 (0.178)
Types of domicile address	local	2386 (60.512)
	intra-city mobility	5 (0.127)
	inter-city mobility (intra-provincial mobility)	250 (6.340)
	inter-provincial mobility	1302 (33.021)
Patient sources	referral	2693 (68.298)
	tracking	800 (20.289)
	see a doctor directly	170 (4.311)
	health check-up	1 (0.000)
	others	279 (7.076)
Case types	new cases	3627 (91.986)
	recurrent cases	316 (8.014)

### 3.2 Time distribution of new PTB

**Fig 1:**
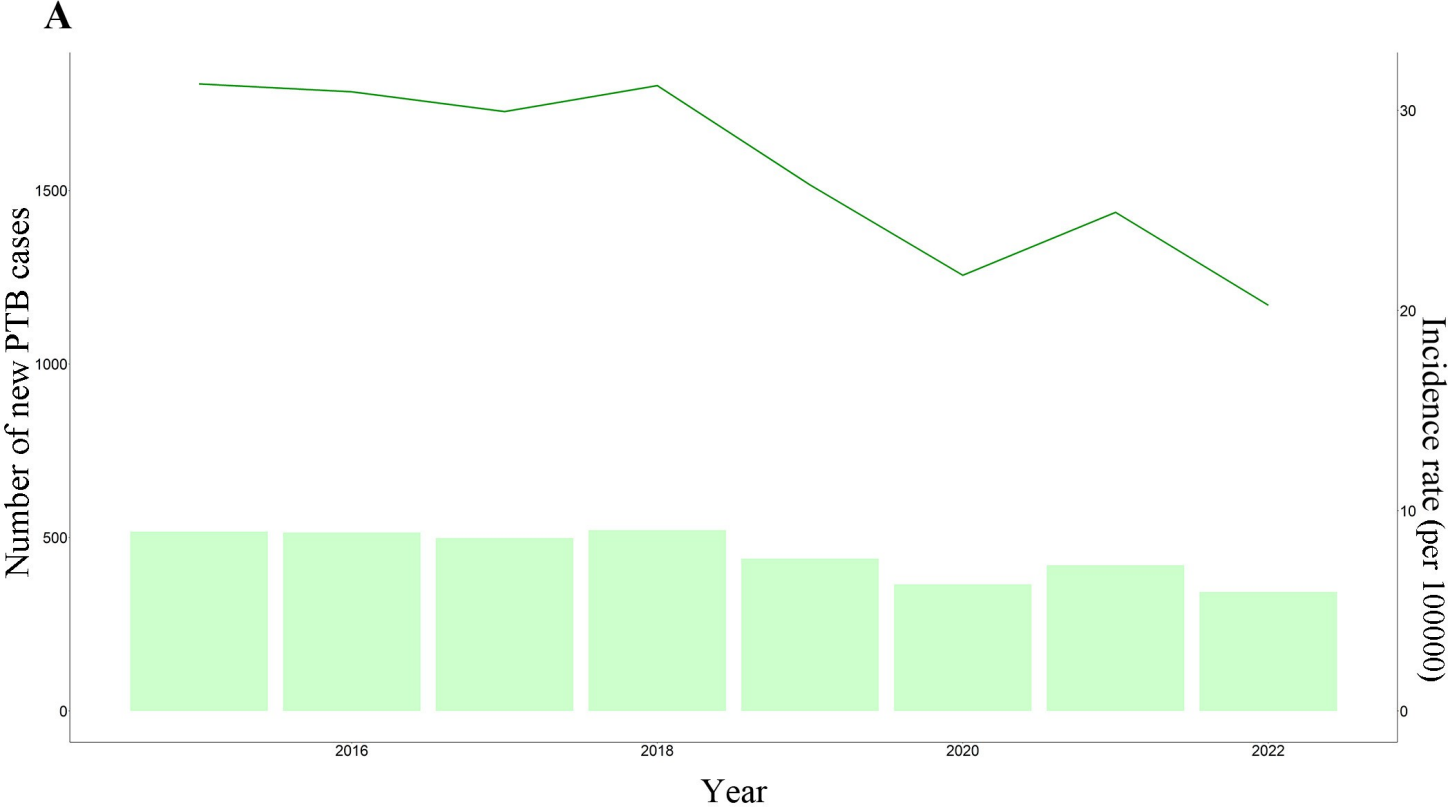
Annual incidence rate and number of cases of new PTB in Changshu city from 2015 to 2022.

**Fig 2:**
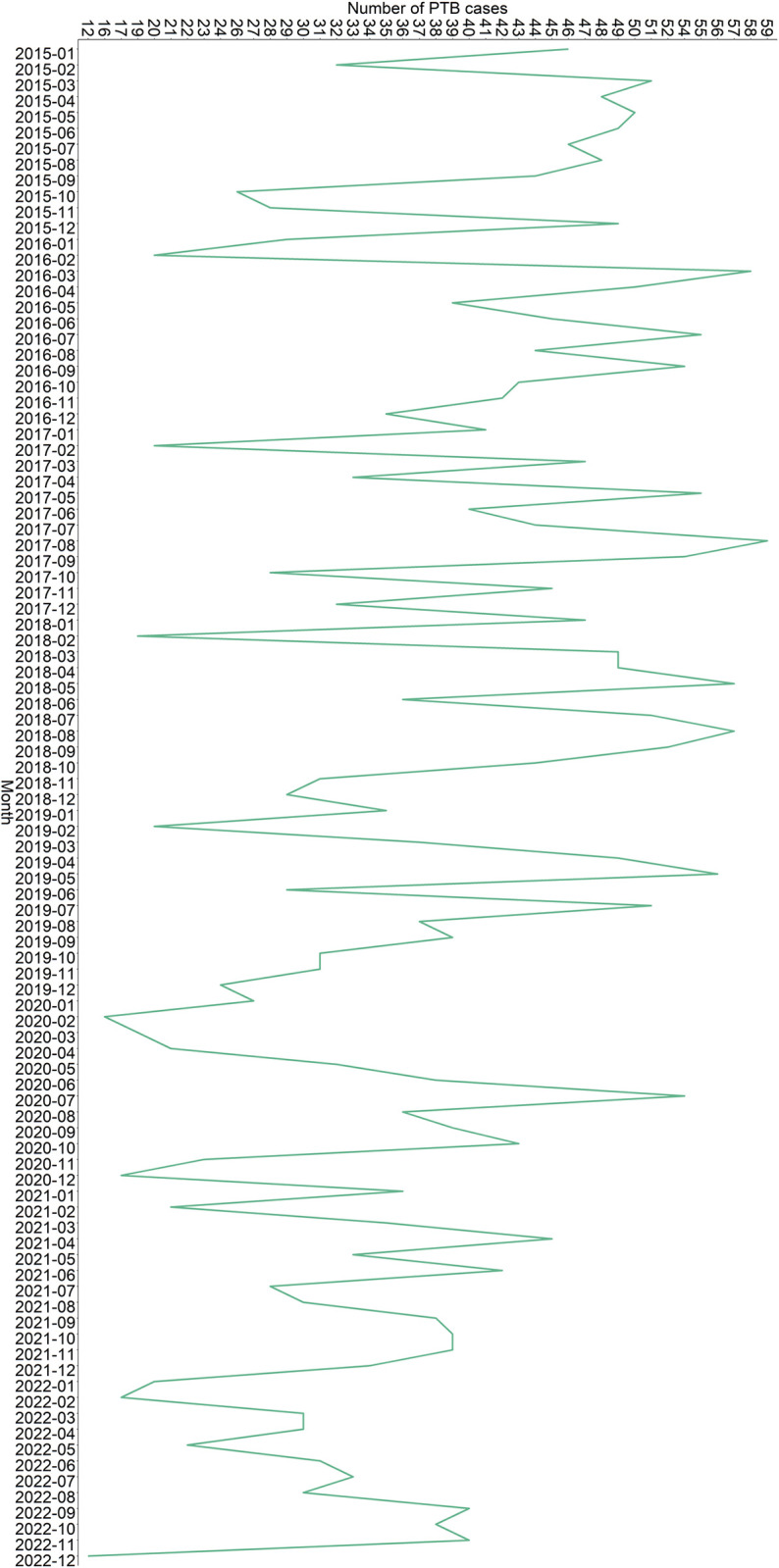
Monthly cases of new PTB in Changshu city from 2015 to 2022.

The average annual new PTB incidence rate from 2015 to 2022 was 27.081 per 100,000. As shown in **Fig 1**, the annual incidence of TB showed an overall trend of downward. The number of new PTB cases in 2018 was 521 with an incidence rate of 31.239 per 100,000, while the number of new PTB cases in 2022 was 343 with an incidence rate of 20.267 per 100,000 (**S1 Table**). The month with the lowest number of new TB cases was December 2022, and the month with the highest number of new PTB cases was August 2017 as shown in **Fig 2**.

### 3.3 Spatial distribution of new PTB

**Fig 3:**
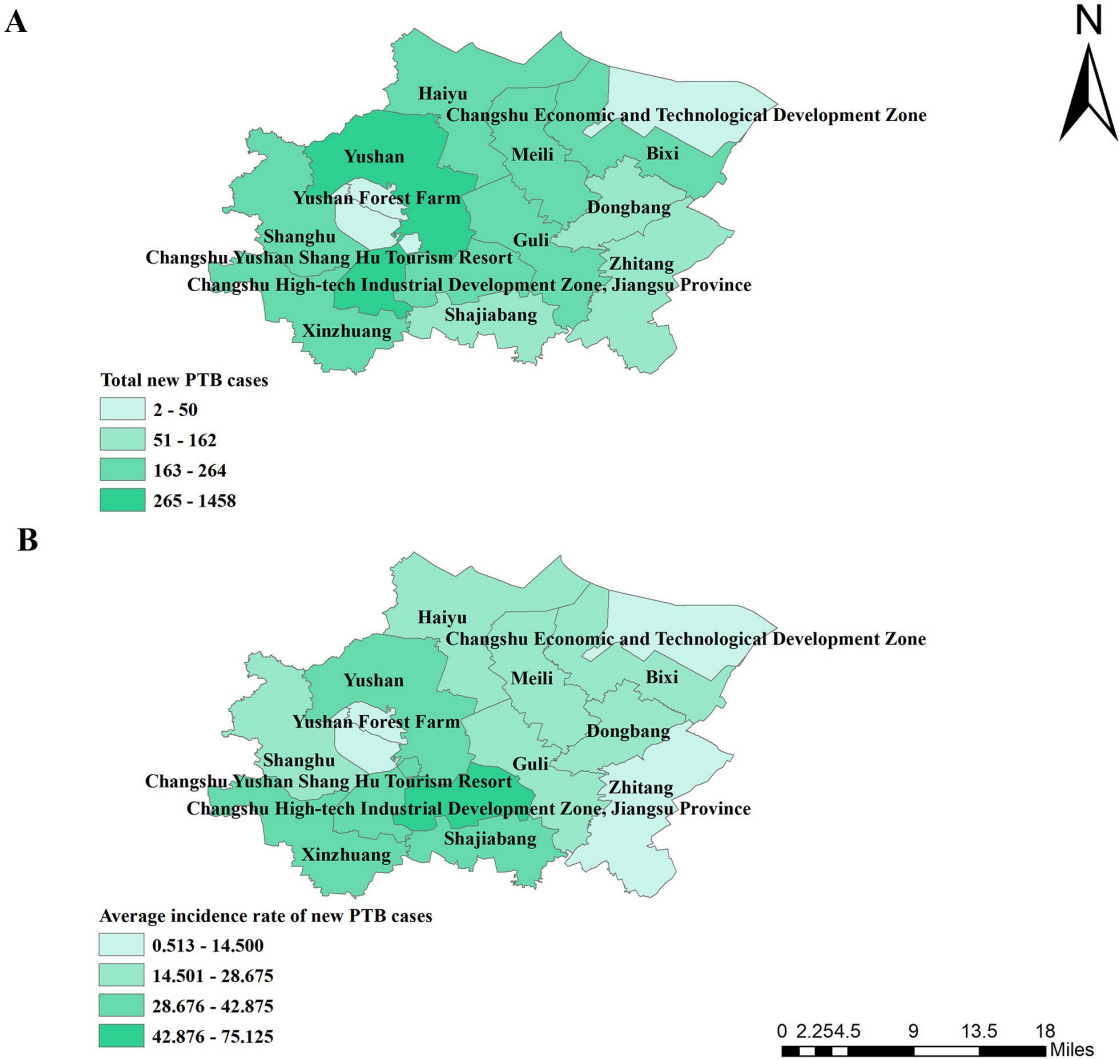
Spatial distribution of new PTB cases and average incidence rate of new PTB cases in Changshu city from 2015 to 2022. (A): Spatial distribution of new PTB cases in Changshu city from 2015 to 2022; (B): Spatial distribution of average incidence rate of new PTB cases in Changshu city from 2015 to 2022.

**Fig 3A** showed the distributions of new PTB cases in each street. Yushan Street had the highest number of new TB cases (1458), followed by Xinzhuang Town (264), Meili Town (253), and Bixi Street (250) (**S2 Table**). The street with the lowest number of PTB cases was Changshu Economic and Technological Development Zone. In terms of incidence rate, the average incidence rate of new PTB cases in each street was between 0.513 per 100,000–75.125 per 100,000 (**S3 Table**), of which the town with the highest incidence rate was Changshu High-tech Industrial Development Zone in Jiangsu Province (75.125 per 100,000), and the town with the lowest incidence rate was Changshu Economic and Technological Development Zone (0.513 per 100.000) in **Fig 3B**.

### 3.4 Global spatial autocorrelation analysis of new PTB cases’ incidence rate

Global spatial autocorrelation analysis of the incidence rate of new PTB cases in each township in Changshu city showed that the Moran’s I = 0.136, Z = 1.359, P = 0.174, indicating that the incidence rate of new PTB cases in each township had no spatial cluster. Further global spatial autocorrelation analysis of the incidence rate of new PTB cases in each township from 2015 to 2022. In 2018, there was a positive spatial correlation (P<0.05) in each township of Changshu city (**Table 2**).

**Table 2:** Global spatial autocorrelation analysis of new PTB cases’ incidence rate by year.

Year	Moran’s I	Z	P
2015	0.110	1.105	0.269
2016	0.032	0.699	0.484
2017	0.072	0.959	0.337
2018	0.300	2.426	0.015
2019	0.154	1.637	0.102
2020	0.062	0.797	0.425
2021	-0.098	-0.162	0.871
2022	-0.005	0.398	0.691

### 3.5 Local spatial autocorrelation analysis of the incidence rate of new PTB cases

**Fig 4:**
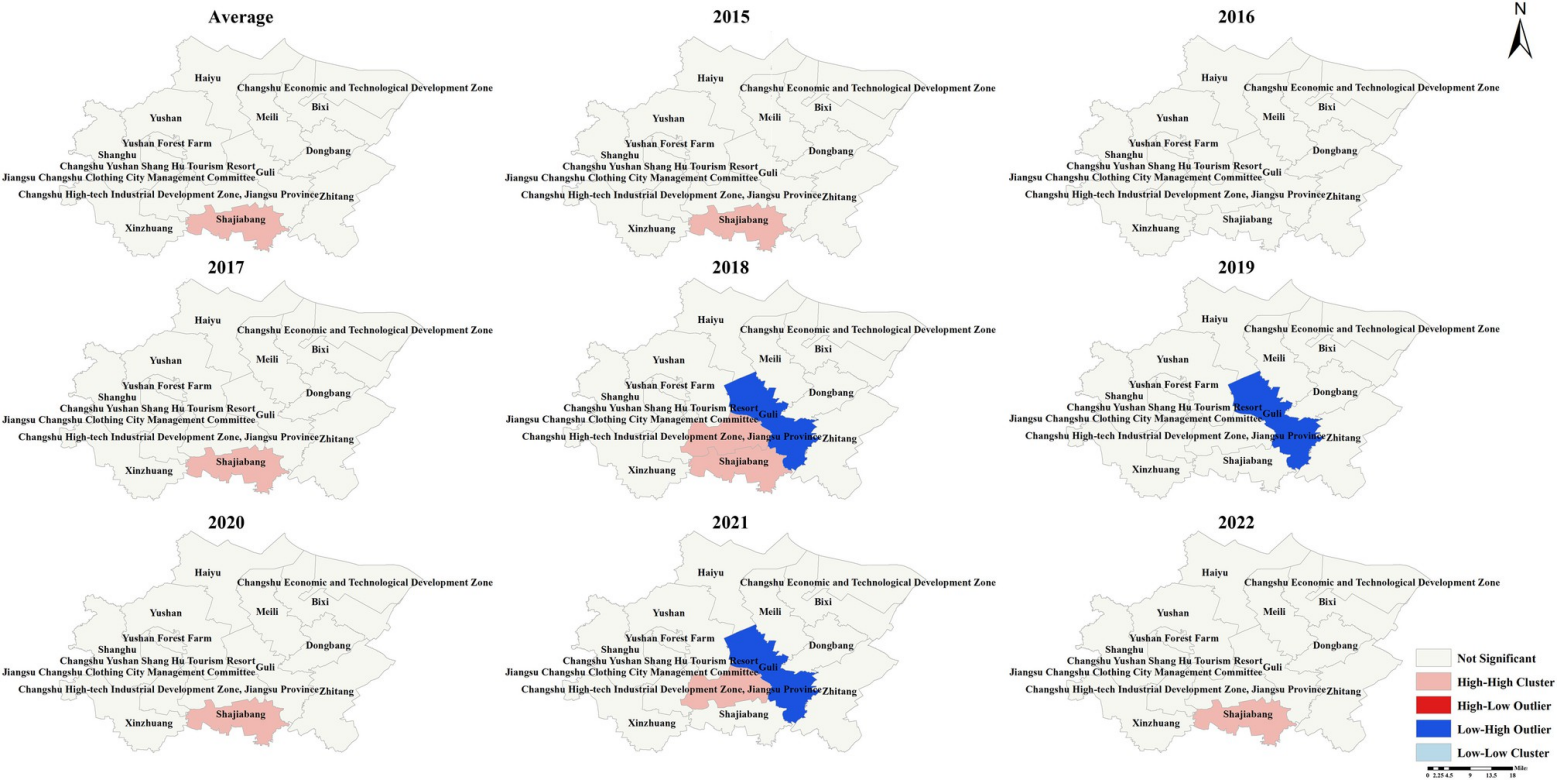
LISA graph of the incidence rate of new PTB cases in Changshu city from 2015 to 2022.

Local spatial autocorrelation analysis of the incidence rate of new PTB cases in each township showed that Shajiabang Town was the cluster area on average. Similar results were observed in 2015, 2017, 2020, and 2022. In addition to the high-high cluster area mentioned above, in 2021, the town of Guli was identified as low-high cluster area. Similarly, Guli town was also the low-high cluster area in 2019. In 2018, Changshu High-tech Industrial Development Zone in Jiangsu Province and Shajiabang town were the high-high cluster areas, while Guli town was identified as the low-high cluster area. There were no clustered townships in Changshu in 2016 (**Fig 4**).

**Fig 5:**
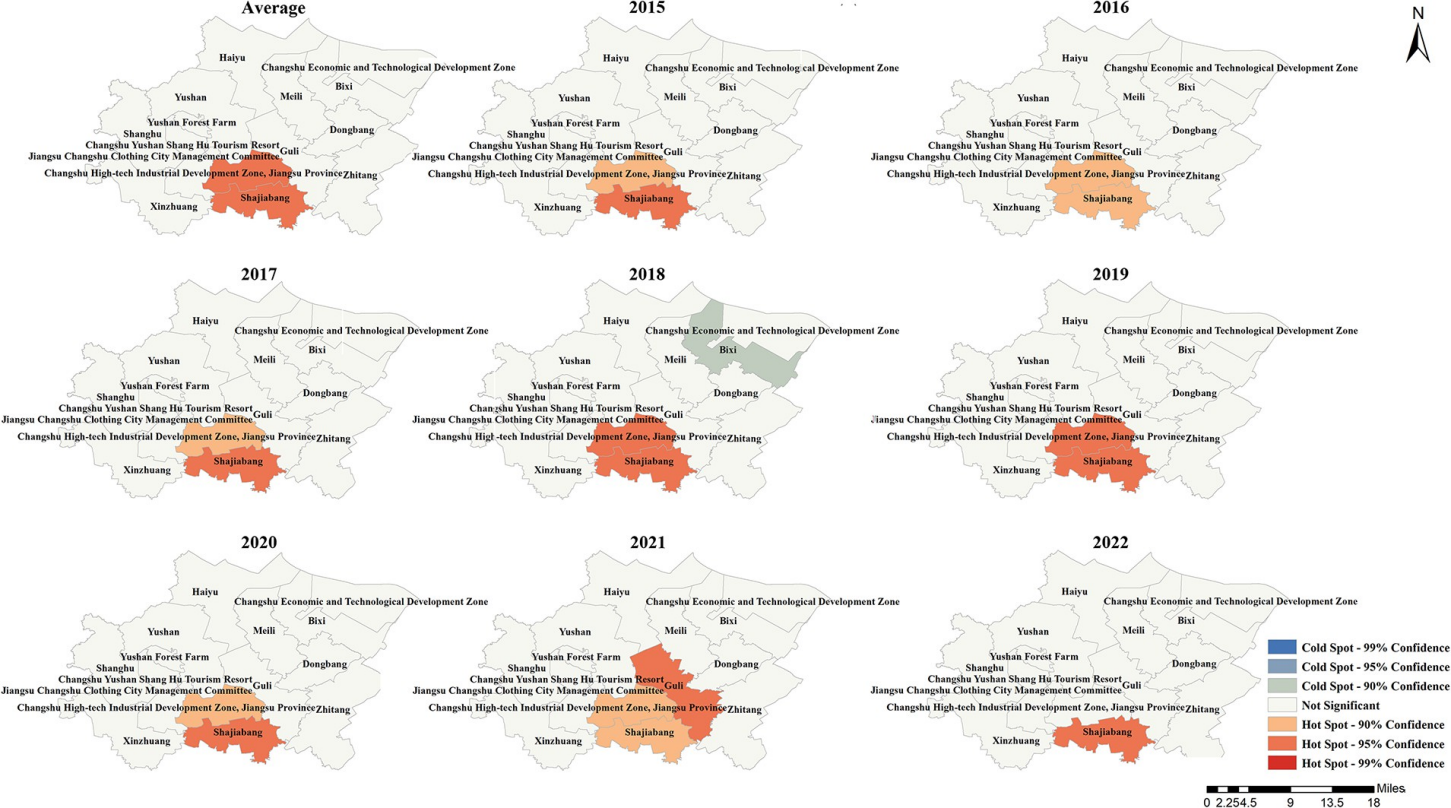
Getis-ord Gi*analysis for the incidence rate of new PTB cases in Changshu city from 2015 to 2022.

According to **Fig 5**, Changshu High-tech Industrial Development Zone in Jiangsu Province and Shajiabang town were the hot spot areas of the incidence rate of new PTB cases in 2018, 2019 and the annual average level, while Shajiabang town was the hot spot area in 2015, 2017, 2020 and 2022. In 2021, Guli town was the only hot spot area among all the townships in Changshu. Note, there were no hot or cold spots in 2016.

### 3.6 Influencing factors for the cure of PTB patients

The above results have reported the incidence of PTB in Changshu. TB, especially multidrug-resistant TB, has posed a health security risk and remains a public health emergency [14]. While many drugs have been used to treat TB, a precise and timely cure is still absent. Hence, the outcome of TB treatment is still unsatisfactory [15]. This study further explored the potential factors that correlated with TB cure outcomes. Patients were divided into two groups (not cured group, cured group) according to the outcome index of whether PTB was cured or not, and the baseline data were compared. As can be seen from **Table 3**, age, diagnostic delay, education, marital status, priority groups, patient source, TB strain types, drug sensitivity, complications, and case types were related factors for the cure of PTB patients (all P<0.05). Further multivariate logistic regression analysis showed that diagnostic delay, MTB, and drug sensitivity were the influencing factors for the cure of PTB patients (**Table 4**).

**Table 3:** Univariate analysis of PTB patients.

Variable		Not cured group	Cured group	Z/χ^2^	P
BMI		21.295 [19.370,23.422]	21.454 [19.482,23.307]	-0.256	0.798
Age		46.000 [28.000,66.000]	51.000 [29.000,68.000]	-3.734	<0.001
Diagnostic delay		22.000 [12.000,42.000]	15.000 [8.000,30.000]	10.965	<0.001
Sex	male	1414 (71.055)	1341 (73.479)	2.788	0.095
	female	576 (28.945)	484 (26.521)		
Education	middle school and below	874 (74.573)	825 (78.947)	6.484	0.039
	senior high school	166 (14.164)	130 (12.440)		
	university or above	132 (11.263)	90 (8.612)		
Marital status	single	423 (26.111)	312 (21.443)	11.355	0.003
	married	1154 (71.235)	1088 (74.777)		
	widowed	43 (2.654)	55 (3.780)		
Alcohol	never	65 (6.348)	73 (7.359)	1.290	0.732
	sometimes	67 (6.543)	64 (6.452)		
	often	97 (9.473)	102 (10.282)		
	everyday	795 (77.637)	753 (75.907)		
Smoke	never	917 (89.638)	899 (90.625)	3.184	0.204
	used to smoke	23 (2.248)	12 (1.210)		
	smoking	83 (8.113)	81 (8.165)		
Key populations	yes	122 (6.131)	159 (8.712)	9.299	0.002
	no	1868 (93.869)	1666 (91.288)		
Patient sources	see a doctor directly	93 (5.118)	73 (4.225)	18.195	<0.001
	referral	1282 (70.556)	1328 (76.852)		
	others	442 (24.326)	327 (18.924)		
Tuberculosis strain types	MTB	167 (60.727)	601 (98.363)	232.687	<0.001
	non-tuberculous mycobacteria	108 (39.273)	10 (1.637)		
Drug sensitivity	sensitivity	213 (72.696)	816 (98.670)	195.524	<0.001
	resistance	80 (27.304)	11 (1.330)		
Comorbidities	with	261 (14.948)	343 (20.688)	19.192	<0.001
	without	1485 (85.052)	1315 (79.312)		
Case types	new cases	1877 (94.322)	1639 (89.808)	26.847	<0.001
	retreat cases	113 (5.678)	186 (10.192)		
Using of FDC	yes	1033 (51.910)	977 (53.534)	1.008	0.315
	no	957 (48.090)	848 (46.466)		
2HRZE4HR	yes	1109 (55.729)	1027 (56.274)	0.115	0.735
	no	881 (44.271)	798 (43.726)		

FDC: Fixed dose compound.

**Table 4:** Multivariate logistic regression analysis on the cured PTB patients.

Predictor		Estimate	SE	Z	P	OR (95%CI)
Intercept		-0.166	1.094	-0.151	0.880	0.847 (0.098–7.430)
Age		-0.013	0.011	-1.206	0.228	0.987 (0.966–1.008)
Diagnostic delay		-0.006	0.002	-2.224	0.026	0.994 (0.989–0.999)
Education	middle school and below					
	senior high school	-0.576	0.488	-1.181	0.238	0.562 (0.218–1.495)
	university or above	0.144	0.692	0.209	0.835	1.155 (0.320–4.995)
Marital status	single					
	married	-0.121	0.528	-0.23	0.818	0.886 (0.299–2.407)
	widowed	1.225	1.14	1.074	0.283	3.405 (0.468–49.379)
Priority groups		0.462	0.607	0.76	0.447	1.587 (0.483–5.331)
Patient sources	see a doctor directly					
	referral	0.865	0.703	1.231	0.218	2.375 (0.524–8.861)
	others	-0.005	0.734	-0.007	0.994	0.995 (0.211–3.955)
Tuberculosis strain types		-3.106	0.511	-6.078	<0.001	0.045 (0.015–0.115)
Drug sensitivity		2.276	0.615	3.698	<0.001	9.739 (3.081–35.829)
Complications		0.030	0.474	0.064	0.949	1.031 (0.421–2.733)
Case types		0.353	0.463	0.762	0.446	1.424 (0.596–3.722)

**Fig 6:**
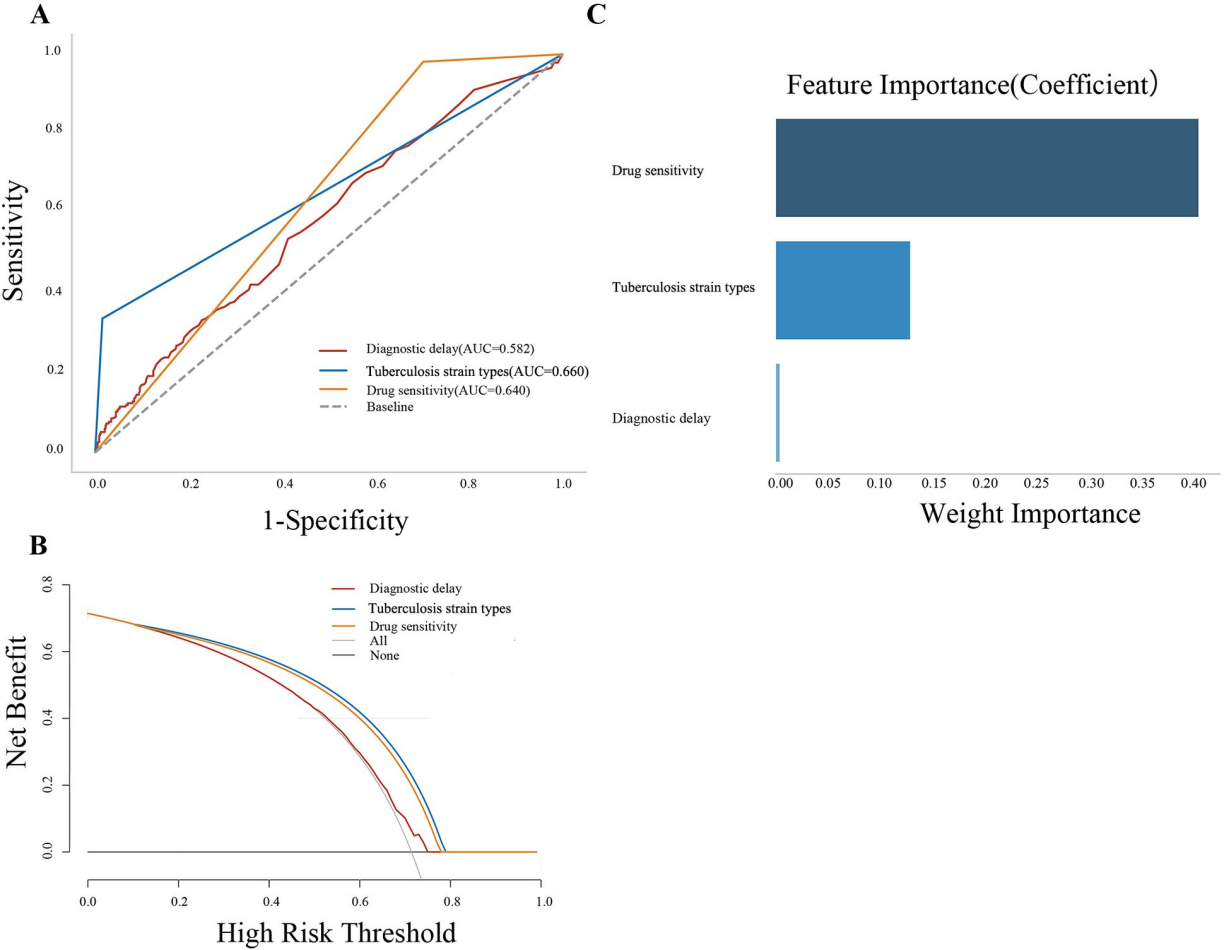
Clinical value analysis of influencing factors in predicting PTB treatment outcome. (A): The ROC curve; (B): The DCA curve; (C): The results of the ranking of factors.

We then evaluated the predictive value of 3 key variables on the PTB treatment outcome by ROC analysis. The AUC of diagnostic delay, TB strain types, and drug sensitivity predicted were 0.582, 0.660, and 0.640, respectively (**Fig 6A**). The results of the AUC comparison of three forecasting methods showed that the predictive effect of TB strain types was higher than the other two indexes, the predictive effect of drug sensitivity was higher than diagnostic delay, and the differences were statistically significant (all P<0.05, **S4 Table**). DCA curve showed that the clinical benefit of the TB strain types and drug sensitivity for predicting the PTB treatment outcome was higher than that of diagnostic delay (**Fig 6B**). Finally, we evaluated the importance of these 3 indexes on the PTB treatment outcome. As can be seen in **Fig 6C**, drug sensitivity ranked first, and diagnostic delay ranked last.

## 4. Discussion

We found that the average annual incidence rate of new PTB cases from 2015 to 2022 was 27.081 per 100,000 in this study. This rate was lower than the incidence rate of PTB patients in China in 2022, but comparable to the average annual reported incidence rate in Shijingshan district (28.60/100,000) and Dongcheng district (28.81/100,000) in Beijing [16,17]. In addition to this, we found that the overall annual incidence rate of PTB showed an overall trend of downward. This trend was consistent with global and national trends [18]. On the one hand, the cause of this phenomenon was closely linked to Corona Virus Disease 2019 (COVID-19). The epidemic situation of COVID-19 seriously affected the diagnosis and treatment of PTB in Jiangsu province. Between January and May 2020, the number of reported PTB cases decreased significantly. Additionally, the completion rate of PTB treatment from 2015 to 2019 was reduced by 10%. Furthermore, in 2020, the screening rate for PTB drug resistance was significantly reduced [19]. On the other hand, the reduction of the annual incidence rate in Changshu city is also the result of strengthened TB prevention and control measures. To reduce the incidence, illness, and mortality rates of TB, as well as to alleviate the burden of TB and improve public health, Jiangsu Province and Suzhou city has issued the "Twelfth Five-Year Plan", "Thirteenth Five-Year Plan", and "Fourteenth Five-Year Plan" for TB control. Therefore, in the post-epidemic era, it is even more necessary to improve the prevention and control of TB and achieve the goal of "stopping tuberculosis" proposed by the World Health Organization in 2035 earlier and better.

Douglas et al. reported for the first time in 1996 that TB had a unique summer peak [20]. There was a peak of TB in summer in Wuhan [21], Birmingham [22], Netherlands [23], Australia [24], and Iran [25]. Borgdorff et al. found that the average incubation period from MTB infection to PTB was 15.6 months [26]. Furthermore, there was a delay in diagnosis [21,27]. Thus, the rise in the spread of MTB during the winter and spring may result in a peak of reported incidence during the summer [21]. On the one hand, Spring Festival is a traditional festival in China, which will cause Spring Festival travel rush and lead to population mobility. Related studies found that transportation, especially buses and planes, was closely related to the spread of MTB [28,29]. On the other hand, climate was the primary factor that affected the seasonal peak of PTB. A study in Hong Kong also found nonlinear and delayed effects of mean temperature and relative humidity on PTB incidence [30]. One long-time series study found a correlation between the decrease in sunshine in winter and the peak incidence of PTB six months later [22]. Studies conducted in India had shown that the incidence of PTB peaks several months after the low-temperature peak throughout the year [31]. Therefore, it is necessary to prevent the occurrence of PTB and strengthen the monitoring of PTB during the winter and spring.

Global spatial autocorrelation analysis showed no clustering of new PTB incidence rate in Changshu townships, but local spatial autocorrelation analysis showed Shajiabang town and Changshu High-tech Industrial Development Zone in Jiangsu Province not only were the high-high cluster areas and the hotspot areas. Shajiabang town became the high-high cluster area and hot spot area, which was inseparable from its history and tourism. First, Shajiabang town became the center of SuChangtai’s anti-Japanese guerrilla base during the Anti-Japanese War and has many scenic spots related to the anti-Japanese history in Shajiabang Scenic Area. Second, Shajiabang, a modern Beijing opera based on Shajiabang’s revolutionary story, is famous for its popularity. The development of tourism led to an increase in population mobility, which to some extent increased the spread of tuberculin. The reason why Changshu Hi-Tech Development Zone in Jiangsu Province became the high-high cluster area and hot spot was mainly related to its technological and economic development. Advanced technology and rapid economic development had attracted a large influx of talent and labor, resulting in intra- and even inter-provincial population movements. Therefore, in the process of PTB prevention and control, more attention should be paid to the central and southern areas of Changshu.

In this paper, we found that short diagnosis delay days promoted the cure of PTB. The independent risk factors for untreated PTB in smear-positive patients in Yunnan Province were low income, lack of medical insurance, and diagnosis delay days of more than 30 days [32]. Other studies had also found that the diagnosis delay was a risk factor for untreated PTB [33,34]. The diagnosis delay may be related to the awareness rate of PTB. Studies found that the awareness rate of information on TB among China residents was low [35,36]. Besides, the presence of MTB also promoted the cure of PTB. Overall, compared with the non-tuberculous mycobacterium, the clinical presentation of MTB was not very hidden, and symptoms were milder. We also found that drug sensitivity was a risk factor. Drug resistance can be categorized as multidrug-resistant (MDR), pan-drug resistant, extensively-drug resistant, and common drug-resistant bacteria include methicillin-resistant Staphylococcus aureus, vancomycin-resistant Enterococcus and so on [37]. Among drug-resistant TB are classified as mono-resistant TB, poly-resistant TB, MDR-TB, pre-extensively drug-resistant TB, extensively drug-resistant TB, and rifampicin-resistant TB [38]. The continuous evolution of MTB has facilitated the emergence of drug-resistant strains and the emergence of increasingly MDR-TB and extensively drug-resistant TB cases [39]. MDR-TB is primarily unresponsive to both isoniazid and rifampicin, the two most effective first-line TB drugs. It was found that the bactericidal ability of the drug, and the resistance of MTB have a close correlation with the peak concentration of the drug as well as the area under the curve and that the peak concentration of the drug that does not reach the minimum inhibitory concentration will rapidly amplify the resistance of the bacteria, thus reducing the bactericidal effect of the drug [40,41]. In many experiments, it had been found that drug resistance was one of the ultimate predictors of unsuccessful mid-term treatment or treatment outcome results [42–44]. We suspected it may be related to patients’ compliance with medication. In this study, the cured group was older, and patients aged >50 years were found to have lower compliance in related studies [45], and the cured group had lower educational qualifications, with lower educational qualifications also affecting patient compliance [46].

In a word, the incidence of PTB in Changshu is still challenging, and there is still a long way to go to control PTB. The incidence of local PTB undoubtedly aggravated the medical and health and economic burden. Health units in Changshu should continue to increase the screening of PTB, take the central and southern regions as the key tracking areas, and completely eliminate the areas where PTB is concentrated; strengthen the publicity and education of people’s awareness of PTB, popularize the knowledge of prevention and control of PTB, and do a good job in regular disinfection in public places and crowded places. Individuals should do a good job in their own hygiene, ventilate frequently, wash their hands frequently, wear masks when going out, and have regular physical examinations [47].

For advantages, in this study, the spatial-temporal visualization of epidemiological data was applied, and the spatial variation or spatial-temporal variation of disease risk was visualized on the map, which provided clues for further etiological research and other research. Our research had some limitations. Firstly, we were unable to conduct a detailed study in a smaller area, such as a village, let alone learn about the case of house-hold TB in the high burden areas in Changshu city. Secondly, the study did not take into account the impact of economic factors, and environmental factors on the incidence of PTB.

## 5. Conclusion

This study identified temporal trends and spatial distribution of PTB cases at the township level in Changshu city from 2015 to 2022. The central and southern areas of Changshu were the high-high cluster areas and hot spots for PTB. Shorter diagnosis delay days and MTB promote the cure of PTB, while drug sensitivity was a risk factor for its cure.

## Supporting information

S1 Table Annual incidence of new PTB patients in Changshu, 2015–2022. (DOCX)

S2 Table Annual number of new PTB patients in towns of Changshu, 2015–2022. (DOCX)

S3 Table Annual incidence of new PTB patients in towns of Changshu, 2015–2022. (DOCX)

S4 Table Comparison of prediction performance of three indicators. (DOCX)

S1 File Raw data. (XLS)

S2 File Map copyright. (DOCX)
