# Supplementary material for: Spatial epidemiological analysis based on township scale and analysis of influencing factors of pulmonary tuberculosis cure of Changshu city from 2015 to 2022
Source: PLoS One. 2025 Jan 16;20(1):e0317269. doi: 10.1371/journal.pone.0317269 (PMC11737766; doi:10.1371/journal.pone.0317269)
Supplement: S1 Table — (DOCX) [file pone.0317269.s001.docx]

| **Supplement Table 1** Annual incidence of new PTB patients in Changshu, 2015-2022 | | | |
| --- | --- | --- | --- |
| Year | Population (10000 person) | Number of new PTB cases | Incidence rate (per 100000) |
| 2015 | 165.05 | 517 | 31.324 |
| 2016 | 166.18 | 514 | 30.93 |
| 2017 | 166.32 | 498 | 29.942 |
| 2018 | 166.78 | 521 | 31.239 |
| 2019 | 167.07 | 439 | 26.276 |
| 2020 | 167.72 | 365 | 21.762 |
| 2021 | 168.61 | 420 | 24.91 |
| 2022 | 169.24 | 343 | 20.267 |
