# Supplementary material for: Spatial epidemiological analysis based on township scale and analysis of influencing factors of pulmonary tuberculosis cure of Changshu city from 2015 to 2022
Source: PLoS One. 2025 Jan 16;20(1):e0317269. doi: 10.1371/journal.pone.0317269 (PMC11737766; doi:10.1371/journal.pone.0317269)
Supplement: S2 Table — (DOCX) [file pone.0317269.s002.docx]

| **Supplement Table 2 Annual number of new PTB patients in towns of Changshu, 2015-2022** | | | | | | | | | |
| --- | --- | --- | --- | --- | --- | --- | --- | --- | --- |
| Name | 2015 | 2016 | 2017 | 2018 | 2019 | 2020 | 2021 | 2022 | Total |
| Guli | 37 | 28 | 27 | 28 | 23 | 19 | 24 | 12 | 198 |
| Zhitang | 21 | 27 | 23 | 20 | 19 | 12 | 21 | 10 | 153 |
| Meili | 46 | 23 | 45 | 32 | 22 | 29 | 33 | 23 | 253 |
| Haiyu | 32 | 31 | 34 | 30 | 27 | 28 | 31 | 24 | 237 |
| Dongbang | 15 | 12 | 8 | 16 | 22 | 14 | 22 | 14 | 123 |
| Yushan | 207 | 220 | 195 | 239 | 176 | 134 | 150 | 137 | 1458 |
| Xinzhuang | 43 | 29 | 37 | 33 | 30 | 32 | 24 | 36 | 264 |
| Shajiabang | 19 | 21 | 20 | 27 | 26 | 17 | 20 | 12 | 162 |
| Yushan Forest Farm | 2 | 3 | 4 | 5 | 1 | 0 | 1 | 2 | 18 |
| Changshu High-tech Industrial Development Zone,Jiangsu Province | 22 | 34 | 28 | 26 | 31 | 26 | 24 | 24 | 215 |
| Shanghu | 33 | 35 | 36 | 40 | 21 | 19 | 24 | 17 | 225 |
| Bixi | 31 | 38 | 33 | 18 | 34 | 29 | 39 | 28 | 250 |
| Changshu Yushan Shanghu Tourism Resort | 0 | 0 | 0 | 0 | 1 | 1 | 5 | 2 | 9 |
| Jiangsu Changshu Clothing City Management Committee | 9 | 13 | 8 | 6 | 6 | 5 | 2 | 1 | 50 |
| Changshu Economic and Technological Development Zone | 0 | 0 | 0 | 1 | 0 | 0 | 0 | 1 | 2 |
