# Supplementary material for: Spatial epidemiological analysis based on township scale and analysis of influencing factors of pulmonary tuberculosis cure of Changshu city from 2015 to 2022
Source: PLoS One. 2025 Jan 16;20(1):e0317269. doi: 10.1371/journal.pone.0317269 (PMC11737766; doi:10.1371/journal.pone.0317269)
Supplement: S3 Table — (DOCX) [file pone.0317269.s003.docx]

| **Supplement Table 3 Annual incidence of new PTB patients in towns of Changshu, 2015-2022** | | | | | | | | | |
| --- | --- | --- | --- | --- | --- | --- | --- | --- | --- |
| Name | 2015 (per 100000) | 2016 (per 100000) | 2017 (per 100000) | 2018 (per 100000) | 2019 (per 100000) | 2020 (per 100000) | 2021 (per 100000) | 2022 (per 100000) | Annual average (per 100000) |
| Guli | 29.3 | 22.1 | 21.1 | 21.8 | 17.8 | 15.1 | 19 | 9.4 | 19.45 |
| Zhitang | 15.8 | 20.3 | 17.2 | 14.9 | 14.1 | 9.4 | 16.5 | 7.8 | 14.5 |
| Meili | 44.4 | 22.1 | 42.9 | 30.4 | 20.8 | 23.6 | 26.7 | 18.5 | 28.675 |
| Haiyu | 27.8 | 26.8 | 29.1 | 25.6 | 23 | 21.2 | 23.5 | 18.0 | 24.375 |
| Dongbang | 22.1 | 17.6 | 11.6 | 23.2 | 31.8 | 19.7 | 30.9 | 19.5 | 22.05 |
| Yushan | 41.7 | 44.1 | 38.8 | 47.3 | 34.7 | 30.5 | 34.0 | 30.8 | 37.7375 |
| Xinzhuang | 52 | 34.9 | 44 | 39.1 | 35.4 | 32.2 | 24.1 | 35.8 | 37.1875 |
| Shajiabang | 43.7 | 48.1 | 44.9 | 60.4 | 58 | 30.6 | 35.9 | 21.4 | 42.875 |
| Yushan Forest Farm | 8.6 | 12.8 | 17 | 21.1 | 4.2 | 00.0 | 5.6 | 11.1 | 10.05 |
| Changshu High-tech Industrial Development Zone, Jiangsu Province | 75.9 | 116.7 | 95.4 | 88.2 | 104.9 | 42.3 | 39 | 38.6 | 75.125 |
| Shanghu | 26.9 | 28.4 | 28.4 | 31.4 | 16.4 | 15.2 | 19.1 | 13.4 | 22.4 |
| Bixi | 27.7 | 33.8 | 29.2 | 15.8 | 29.7 | 24.9 | 33.5 | 23.8 | 27.3 |
| Changshu Yushan Shanghu Tourism Resort | 0.0 | 0.0 | 0.0 | 0.0 | 3.4 | 3.5 | 17.6 | 7.0 | 3.9375 |
| Jiangsu Changshu Clothing City Management Committee | 59.5 | 85.6 | 52.3 | 39 | 38.9 | 36.8 | 14.7 | 7.3 | 41.7625 |
| Changshu Economic and Technological Development Zone | 0.0 | 0.0 | 0.0 | 2.1 | 0.0 | 0.0 | 0.0 | 2 | 0.5125 |
