# Supplementary material for: Spatial epidemiological analysis based on township scale and analysis of influencing factors of pulmonary tuberculosis cure of Changshu city from 2015 to 2022
Source: PLoS One. 2025 Jan 16;20(1):e0317269. doi: 10.1371/journal.pone.0317269 (PMC11737766; doi:10.1371/journal.pone.0317269)
Supplement: S4 Table — (DOCX) [file pone.0317269.s004.docx]

| **Supplement Table 4 Comparison of prediction performance of three indicators** | | | |
| --- | --- | --- | --- |
| Name | Diagnostic delay | Tuberculosis strain style | Drug sensitivity |
| Diagnostic delay | | 0.003 | <0.001 |
| Tuberculosis strain style | 0.003 |  | <0.001 |
| Drug sensitivity | <0.001 | <0.001 |  |
