# Supplementary material for: Spatial epidemiological analysis based on township scale and analysis of influencing factors of pulmonary tuberculosis cure of Changshu city from 2015 to 2022
Source: PLoS One. 2025 Jan 16;20(1):e0317269. doi: 10.1371/journal.pone.0317269 (PMC11737766; doi:10.1371/journal.pone.0317269)
Supplement: S2 File — (DOCX) [file pone.0317269.s006.docx]

I request permission for the open-access journal PLOS ONE to publish Figures 2,3 and 4 under the Creative Commons Attribution License (CCAL) CC BY 4.0 (http://creativecommons.org/licenses/by/4.0/).

Below is the review number for the map: GS(2022)1873

Below is the URL for the review number of the map: <http://xzqh.mca.gov.cn/map>.
